# Electro-Haptic Stimulation: A New Approach for Improving Cochlear-Implant Listening

**DOI:** 10.3389/fnins.2021.581414

**Published:** 2021-06-09

**Authors:** Mark D. Fletcher, Carl A. Verschuur

**Affiliations:** ^1^Faculty of Engineering and Physical Sciences, University of Southampton Auditory Implant Service, University of Southampton, Southampton, United Kingdom; ^2^Faculty of Engineering and Physical Sciences, Institute of Sound and Vibration Research, University of Southampton, Southampton, United Kingdom

**Keywords:** vibrotactile, hearing impaired, haptic sound-localization, hearing aid, neuroprosthetic, somatosensory, tactile aid, cross-modal

## Abstract

Cochlear implants (CIs) have been remarkably successful at restoring speech perception for severely to profoundly deaf individuals. Despite their success, several limitations remain, particularly in CI users’ ability to understand speech in noisy environments, locate sound sources, and enjoy music. A new multimodal approach has been proposed that uses haptic stimulation to provide sound information that is poorly transmitted by the implant. This augmenting of the electrical CI signal with haptic stimulation (electro-haptic stimulation; EHS) has been shown to improve speech-in-noise performance and sound localization in CI users. There is also evidence that it could enhance music perception. We review the evidence of EHS enhancement of CI listening and discuss key areas where further research is required. These include understanding the neural basis of EHS enhancement, understanding the effectiveness of EHS across different clinical populations, and the optimization of signal-processing strategies. We also discuss the significant potential for a new generation of haptic neuroprosthetic devices to aid those who cannot access hearing-assistive technology, either because of biomedical or healthcare-access issues. While significant further research and development is required, we conclude that EHS represents a promising new approach that could, in the near future, offer a non-invasive, inexpensive means of substantially improving clinical outcomes for hearing-impaired individuals.

## Introduction

Cochlear implants (CIs) are one of the most successful neuroprostheses, allowing those with severe-to-profound deafness to access sound through electrical stimulation of the cochlea. Over 18,000 people in the United Kingdom alone currently use a CI (Hanvey, 2020), although it has been estimated that only 1 in 20 adults who could benefit from a CI have accessed one (Raine et al., 2016). Despite the success of CIs, there remain significant limitations in the performance that can be achieved by users (Spriet et al., 2007; Dorman et al., 2016). Recently, however, a new multimodal approach to improve CI user performance has emerged (Huang et al., 2017; Fletcher et al., 2018, 2019, 2020a, 2020b, 2020c; Ciesla et al., 2019; Fletcher, 2020; Fletcher and Zgheib, 2020). This approach uses “electro-haptic stimulation” (EHS)^1^, whereby the electrical CI signal is augmented by haptic stimulation, which provides missing sound-information. In addition to augmenting CI listening, new advances in haptic technology mean that haptic stimulation could provide a low-cost means to aid the many millions of people worldwide with disabling hearing loss who cannot access CI technology. In the following three sections of this review, we first examine the evidence of EHS benefits to CI listening, before reviewing the potential for a new generation of haptic aids to support those who are unable to access hearing-assistive devices. Finally, we discuss key areas in which further research is required, such as in identifying the optimal signal-processing regime to maximize EHS benefit, establishing the effects of long-term training with EHS, and understanding the mechanisms that underlie to EHS benefit.

## Electro-Haptic Stimulation

**FIGURE 1:**
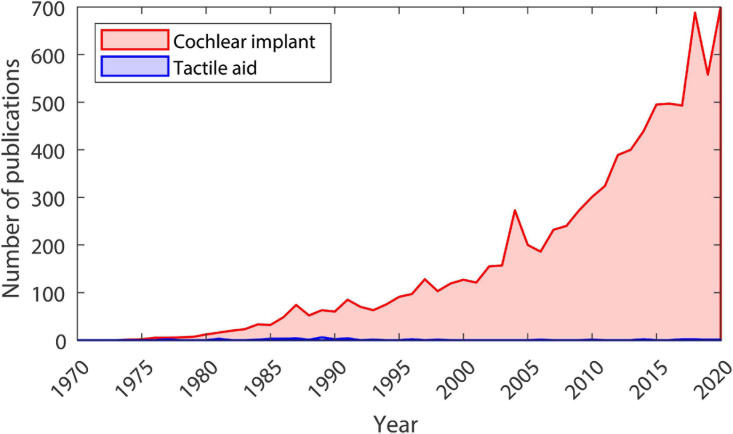
Number of publications each year from 1970 to 2020. Data taken from Google Scholar searches for articles (including patents, not including citations) with the term “tactile aid” (shown in blue) or “cochlear implant” (shown in red) in the title. The search was conducted on 07/02/2021.

In the 1920s, the first “tactile aids” were developed to assist profoundly deaf children in the classroom (Gault, 1924, 1930). This was followed by influential work, beginning in the late 1960s, where visual information was delivered to blind individuals using haptic stimulation on the finger or back. Participants were able to recognize faces, complete complex inspection-assembly tasks, and judge the speed and direction of a rolling ball (Bach-y-Rita et al., 1969, 2003; Bach-y-Rita, 2004). Fascinatingly, after training, participants reported that objects became externalized, seeming as though they were outside of their body rather than being located on the skin (Bach-y-Rita, 2004). In the 1980s and 1990s, largely due to technological advances, interest in using tactile aids to treat deafness grew substantially. In the mid-1980s, one study showed that it was possible to learn a vocabulary of 250 words with a tactile aid (Brooks et al., 1985). This included the ability to discriminate words that differ only by place of articulation, such as “so” and “show” or “let” and “net.” Another set of studies showed that, for both hearing and post-lingually deafened individuals who are lip reading without auditory cues, haptic stimulation can increase the percentage of words recognized within a sentence by more than 15% (De Filippo, 1984; Brooks et al., 1986a; Hanin et al., 1988; Cowan et al., 1991; Reed et al., 1992). However, the development of tactile aids was halted by dramatic improvements in CI technology, which allowed users to achieve speech recognition far better than could conceivably be achieved using a tactile aid (Zeng et al., 2008). By the late 1990s, the use and development of tactile aids had almost completely ceased, and a rapid expansion in CI research began (see Figure 1).

In recent decades, while the expansion of CI research has continued, the pace of improvements in patient outcomes has slowed (Zeng et al., 2008; Wilson, 2015). Despite the huge success of CIs, there remain significant limitations for even the best-performing users (Wilson, 2017), as well as substantial variation in performance between individuals (Tamati et al., 2019). For example, CI users have limited pitch perception (D’Alessandro and Mancini, 2019), frequency resolution (O’Neill et al., 2019), and dynamic range (Bento et al., 2005). These issues in extracting basic sound properties translate into limitations in real-world listening, with CI users often struggling to understand speech in challenging listening conditions (Hazrati and Loizou, 2012), struggling to locate sounds (Dorman et al., 2016), and having substantially reduced music appreciation (McDermott, 2004; Dritsakis et al., 2017). For CI users with useful residual acoustic hearing, combining electrical CI stimulation with acoustic stimulation (electro-acoustic stimulation) has been shown to improve performance (O’Connell et al., 2017). Impaired acoustic hearing can transmit important missing sound-information, such as pitch, temporal fine structure, and dynamic changes in intensity, more effectively than a CI (Gifford et al., 2007; Gifford and Dorman, 2012). However, the proportion of CI users with useful residual acoustic hearing is small (Verschuur et al., 2016) and residual hearing deteriorates at a faster rate after implantation (Wanna et al., 2018).

Electro-haptic stimulation has recently emerged as an alternative approach to improve CI outcomes. EHS uses haptic stimulation to augment the CI signal, rather than as an alternative to CI stimulation, as was the case with tactile aids. Early evidence suggests that EHS can improve speech-in-noise performance, sound localization, and music perception in CI users. Two recent studies showed improved speech-in-noise performance when the fundamental frequency (*F_0*) of speech (an acoustic correlate of pitch) was presented through haptic stimulation on the finger. This was demonstrated both for CI users (Huang et al., 2017) and for normal-hearing participants listening to simulated CI audio (Ciesla et al., 2019). However, in these studies, the haptic signal was extracted from the clean speech signal, which would not be available in the real world.

**FIGURE 2:**
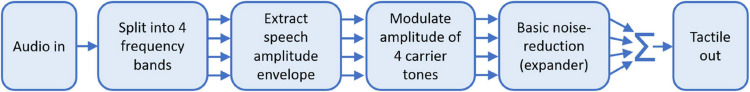
Block diagram describing the haptic signal-processing strategy used by Fletcher et al. (2019).

Fletcher et al. (2019) showed that presenting the speech amplitude envelope through haptic stimulation also improves speech-in-noise performance in CI users. In this study, the haptic signal was extracted from the speech-in-noise signal using a simple noise-reduction technique. Furthermore, the signal processing used could be applied in real-time on a compact device and haptic stimulation was delivered to the wrist, which is a more suitable site for a real-world application. A block diagram of the signal-processing strategy used is shown in Figure 2. The amplitude envelope is extracted from the audio in four frequency bands, which cover the frequency range where speech energy is maximal. Each of the four envelopes is then used to modulate the amplitude of one of four carrier tones. The carrier tone frequencies are focused where tactile sensitivity is highest and are spaced so that they are individually discriminable. Each tone is then passed through an expander, which exaggerates larger amplitude modulations and acts as a basic noise-reduction strategy. The tones are then delivered to each wrist through a single shaker contact. Using this approach, participants were able to recognize 8% more words in multi-talker noise with EHS compared to with their CI alone, with word recognition for some participants increasing by more than 20%. Similar benefit to speech-in-noise performance has also been found in normal-hearing participants listening to simulated CI audio (Fletcher et al., 2018). This study used a similar haptic signal-processing strategy, but haptic stimulation was delivered to the fingertip rather than the wrist.

In addition to these EHS studies, which used co-located speech and noise sources, Fletcher et al. (2020b) has shown large benefits of EHS for spatially separated speech and noise in unilaterally implanted CI users. In this study, the audio received by devices behind each ear was converted to haptic stimulation on each wrist. A similar signal-processing strategy to Fletcher et al. (2019) was used, but without the expander. EHS was found to improve speech reception thresholds in noise by 3 dB when the speech was presented directly in front and the noise was presented either to the implanted or non-implanted side. This improvement is comparable to that observed when CI users use implants in both ears rather than one (van Hoesel and Tyler, 2003; Litovsky et al., 2009; see Fletcher et al., 2020b for discussion). Interestingly, no improvement in speech-in-noise performance with EHS was observed when the speech and noise were co-located. This indicates that the expander was critical to achieving the performance enhancement measured by Fletcher et al. (2019).

In addition to work showing benefits to speech-in-noise performance, EHS has also been shown to substantially improve sound localization in CI users (Fletcher and Zgheib, 2020; Fletcher et al., 2020a). Like in Fletcher et al. (2020b), in these studies the speech amplitude envelope was extracted from audio received by hearing-assistive devices behind each ear and delivered through haptic stimulation on each wrist. Remarkably, using this approach, unilaterally implanted CI users were able to locate speech more accurately than bilateral CI users and at a comparable accuracy to bilateral hearing-aid users (Fletcher et al., 2020a). Furthermore, participants were found to perform better when audio and haptic stimulation were provided together than when either was provided alone. This suggests that participants were able to combine audio and haptic information effectively. Another study used a more sophisticated signal-processing strategy, which included individual correction for differences in tactile sensitivity, and gave extensive training (Fletcher and Zgheib, 2020). Using this approach, still greater haptic sound-localization accuracy was achieved and performance was found to improve continuously throughout an extended training regime.

Another recent set of studies have shown evidence that haptic stimulation might enhance music perception in CI users. Haptic stimulation on the fingertip (Huang et al., 2019) or wrist (Luo and Hayes, 2019) was found to improve melody recognition. In both these studies, haptic stimulation was delivered via a single motor. For stimulation on the fingertip, the low-frequency portion of the audio signal was delivered. For stimulation on the wrist, the *F_0* of the audio was extracted and delivered through changes in the amplitude and frequency of the haptic signal, which varied together. This latter approach precludes the presentation of intensity information. In another study, intensity information was delivered through intensity and frequency variations and *F_0* information was delivered through changes in the location of stimulation along the forearm (Fletcher et al., 2020c). The mosaicOne_B device used in this study also incorporates a new noise-reduction strategy for *F_0* extraction. To assess the effectiveness of the mosaicOne_B, pitch discrimination was measured with and without background noise. On average, participants were able to discriminate sounds whose *F_0* differed by just 1.4%. This is less than a semitone, which is the minimum pitch change in most western melodies, and is substantially better than is typically achieved by CI users (Kang et al., 2009; Drennan et al., 2015). In addition, pitch discrimination was found to be remarkably robust to background noise. Even when the noise was 7.5 dB louder than the signal, no reduction in performance was observed and some participants were still able to achieve pitch-discrimination thresholds of just 0.9%. It should be noted, however, that inharmonic background noise was used. Further work is required to establish the effectiveness of this approach for delivering pitch information when other harmonic sounds are also present, as is common in music and real-world listening scenarios. Future studies should also assess whether the mosaicOne_B can be used to enhance speech-in-noise performance.

While early evidence of EHS benefit to CI listening is highly promising, there are two key issues that should be addressed to fully assess its potential. Firstly, how effective is the tactile system at transferring sound information and, secondly, to what extent are haptic and CI signals linked together in the brain? These issues will be discussed in the following sections.

### Can the Tactile System Effectively Transfer Sound Information?

#### Limits of the Tactile System

When assessing the potential of EHS and when designing haptic devices, it is important to understand the limits of the tactile system in transferring intensity, time, and frequency information. The tactile system is known to be highly sensitive to intensity differences. The just-noticeable intensity difference between two successive stimuli on the hand or index finger is around 1.5 dB (Craig, 1972; Gescheider et al., 1996b) and there is evidence that sensitivity is similar, or perhaps even greater, on the wrist (Summers et al., 2005). This sensitivity to intensity differences is comparable to that of the healthy auditory system (Harris, 1963; Penner et al., 1974; Florentine et al., 1987). When assessing the capacity of the tactile system to deliver intensity information, it is also important to consider its dynamic range and the number of discriminable intensity steps it contains. This determines how well the system can portray absolute intensity information, as well as how large a difference between stimuli it can represent. The dynamic range for electrical CI stimulation is around 10–20 dB (Zeng and Galvin, 1999; Zeng et al., 2002). The dynamic range of the tactile system at the fingertip or wrist, however, is around four times larger (∼60 dB; Verrillo et al., 1969; Fletcher et al., 2021a, b). Across the dynamic range, approximately 40 intensity steps can be discriminated with haptic stimulation (Gescheider et al., 1996b), whereas CI users can discriminate around 20 intensity steps (Kreft et al., 2004; Galvin and Fu, 2009). Given the high sensitivity to intensity differences and large dynamic range, the tactile system seems well suited to providing supplementary sound intensity information for CI users.

In contrast to intensity sensitivity, the temporal precision of the tactile system is more limited than for CI users. Temporal precision of CI stimulation is high, with gap detection thresholds typically 2–5 ms in CI users (Moore and Glasberg, 1988; Garadat and Pfingst, 2011), which is similar to normal-hearing listeners (Plomp, 1964; Penner, 1977). For haptic stimulation, however, gap detect thresholds are ∼10 ms (Gescheider, 1966, 1967). The tactile system is also more susceptible to masking from stimuli that are temporally remote. Masking sounds that precede a signal by 100 ms or more typically do little masking for normal-hearing listeners (Elliot, 1962) or for CI users (Shannon, 1990). However, for haptic stimulation, some masking continues even if the masker precedes the signal by several hundreds of milliseconds (Gescheider et al., 1989).

In addition to having limited temporal precision, the tactile system is poor at discriminating stimulation at different frequencies. The healthy auditory system can detect frequency changes of just 1% at 100 Hz and 10% at 10 kHz (Moore, 1973). CI users are much poorer at frequency discrimination, being able to detect minimum frequency changes of ∼10–25% at 500 Hz and ∼10–20% at 4 kHz (Turgeon et al., 2015). The tactile system is poorer still, only able to detect changes of ∼20% at 50 Hz and of ∼35% at 200 Hz for stimulation on the finger (Goff, 1967) or forearm (Rothenberg et al., 1977).

The properties of the tactile system detailed above focus mainly on the finger, hand, wrist, or forearm (where most data are available). However, tactile aids have previously been mounted at various points around the body, including the sternum (Blamey and Clark, 1985), abdomen (Sparks et al., 1978), and back (Novich and Eagleman, 2015). Tactile sensitivity is known to vary markedly across body sites (e.g., Wilska, 1954). This is partly due to the different receptors and structure of glabrous (smooth) skin and non-glabrous (hairy) skin (Bolanowski et al., 1994; Cholewiak and Collins, 2003). Relatively few studies have compared sensitive across sites. The available data suggest that sensitivity is highest at the fingertip and reduces with distance from the finger, at the palm, wrist, forearm, and biceps (Wilska, 1954; Verrillo, 1963, 1966, 1971; Cholewiak and Collins, 2003; Fletcher et al., 2021b). The sternum has been found to be approximately as sensitive as the forearm, with areas of the back being less sensitive, and the abdomen being less sensitive still (Wilska, 1954).

#### Transfer of Complex Sound Information

The aim of EHS is to use haptic stimulation to deliver important auditory cues that are not well perceived through a CI. For CI users, the amplitude envelope is particularly important, as spectral information is severely degraded and so cannot be fully utilized (Blamey and Clark, 1990). The amplitude envelope facilitates the segmentation of the speech stream and the separation of speech from background noise (by marking syllable and phonemic boundaries over time and giving information about syllable stress and number; Kishon-Rabin and Nir-Dankner, 1999; Won et al., 2014; Cameron et al., 2018). However, the coding of amplitude envelope information by the CI is highly susceptible to degradation both by external factors, such as background noise (Chen et al., 2020), and by internal factors, such as the limited dynamic-range available through electrical stimulation (see previous section) and the interaction between electrode channels (Chatterjee and Oba, 2004).

The tactile system is well suited to providing amplitude envelope information. In addition to having a much larger dynamic range than electrical CI stimulation, the tactile system is highly sensitive to amplitude envelope differences across the range of modulation frequencies most important for speech recognition (Weisenberger, 1986; Drullman et al., 1994). Interestingly, there is evidence that the wrist (a site commonly used for haptic devices) is particularly sensitive to amplitude modulation (Summers et al., 1994, 2005). Because of this high sensitivity and the importance to speech perception, some tactile aids (Proctor and Goldstein, 1983; Spens and Plant, 1983) and EHS approaches (Fletcher et al., 2018, 2019, 2020b) have focused on the provision of amplitude envelope information.

A further crucial limitation for CI users is the poor transmission of pitch information, particularly for speech and music (McDermott, 2004; Chatterjee and Peng, 2008). Accurate coding of pitch information in speech (through *F_0* or its harmonics) is required for perception of supra-segmental and paralinguistic information, including intonation, stress, and identification of talker mood or identity (Traunmuller, 1988; Murray and Arnott, 1993; Summers and Gratton, 1995; Most and Peled, 2007; Meister et al., 2009). Pitch also serves as an important cue for talker segregation in noisy listening environments (Leclere et al., 2017). However, *F_0* changes in speech over time or between talkers are not well coded by CIs. This is because the *F_0* for speech typically varies within the frequency range coded by a single CI electrode, preventing the use of across-electrode pitch cues (Swanson et al., 2019; Pisanski et al., 2020).

**FIGURE 3:**
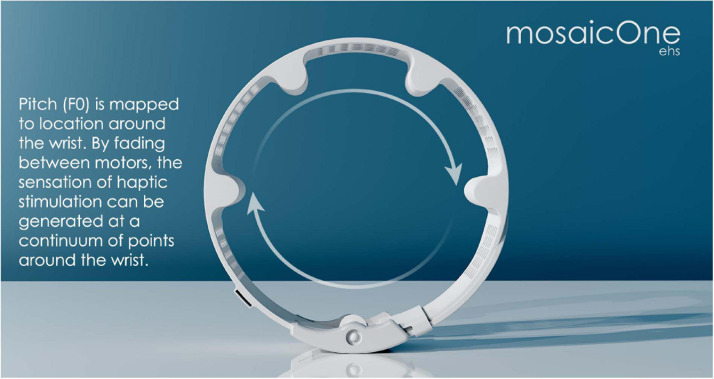
Image of the mosaicOne_C device currently being developed at the University of Southampton as part of the Electro-Haptics Project. Text and arrows highlight that the device has four motors (extruding from the wristband), which are faded between to create the sensation of haptic stimulation at continuum of points around the wrist. Image reproduced with permission of Samuel Perry and Mark Fletcher.

The tactile system is poor at transferring information through changes in stimulation frequency. Nonetheless, some EHS approaches have used stimulation frequency to deliver spectral (Fletcher et al., 2018, 2019, 2020b) or *F*_0_(Huang et al., 2017) information. An alternative approach has been used by some tactile aids (Brooks and Frost, 1983; Hanin et al., 1988) and the mosaicOne series of EHS devices (Fletcher, 2020; Fletcher et al., 2020c). In these devices, frequency or pitch information is transferred through changes in the location of stimulation either along the forearm or around the wrist (see Figure 3).

Amplitude envelope and *F_0* information have been shown to facilitate similar levels of speech recognition in quiet when provided through either haptic (Grant et al., 1985) or auditory stimulation (Summers and Gratton, 1995). These cues have also been found to provide similar benefit to speech-in-noise performance for CI users when provided through haptic (Huang et al., 2017; Fletcher et al., 2019, 2020b) or auditory (Brown and Bacon, 2009) stimulation. However, providing both amplitude envelope and *F_0* cues together has been shown to facilitate better speech recognition than providing either alone, as each provides different information (Summers and Gratton, 1995; Brown and Bacon, 2009).

Another auditory feature that is important to speech recognition is spectral shape (Guan and Liu, 2019). Accurate perception of spectral shape is critical for phoneme recognition as it provides information about the place of articulation for consonants and the identity of vowels (Kewley-Port and Zheng, 1998; Li et al., 2012). Although CI users are able to access gross spectral information, perception of spectral shape and corresponding phoneme identification abilities are limited compared to normal-hearing listeners (Sagi et al., 2010). Future EHS approaches might therefore enhance speech perception in CI users by providing access to information about spectral shape, such as flatness, spread, or centroid. Currently, EHS devices like the mosaicOne_C provide amplitude envelope and *F_0* information using a one-dimensional array of haptic stimulators (with amplitude encoded as simulation intensity and *F_0* coded to location along the array). Some tactile aids used two-dimensional arrays (typically coding sound intensity on one dimension and frequency on the other; Sparks et al., 1978; Snyder et al., 1982). This two-dimensional array approach could be used to extend existing EHS devices and allow for coding of additional spectral sound features.

Finally, CI users tend to have limited access to cues that are critical to sound localization and segregation, such as time and intensity differences across the ears (van Hoesel and Tyler, 2003; Litovsky et al., 2009; Dorman et al., 2016). This is primarily because the majority of adult CI users are implanted in only one ear (Raine, 2013), but CI users implanted in both ears also have substantially limited spatial hearing (Dorman et al., 2016). This is due to the fact that timing differences between the ears cannot be accessed or are highly degraded (Laback et al., 2004) and so bilaterally implanted CI users rely primarily or entirely on intensity differences (van Hoesel and Tyler, 2003). These intensity differences can be heavily distorted by independent pre-processing between devices (particularly automatic gain control; Potts et al., 2019). Additional factors that limit spatial hearing abilities in bilateral CI users are mismatches across devices in the perceived intensity and the place of electrical stimulation within the cochlea (Kan et al., 2019) as well as the impaired perception of spectral (e.g., pinna) cues (Fischer et al., 2020).

Previous EHS studies have used haptic stimulation to provide spatial-hearing cues to CI users. In these studies, the audio received by devices behind each ear was converted to haptic stimulation on each wrist. This meant that time and intensity differences across the ears were available as across-wrist time and intensity differences. Using this approach, large improvements were shown in both sound-localization accuracy (Fletcher and Zgheib, 2020; Fletcher et al., 2020a) and speech reception for spatially separated speech and noise (Fletcher et al., 2020b). Two recent studies have investigated sensitivity to across-wrist tactile time and intensity differences (Fletcher et al., 2021a, b). Encouragingly, participants could detect tactile intensity differences across the wrists of just 0.8 dB, which is similar to (or perhaps even better than) sensitivity to sound intensity differences across the ears (Grantham, 1984). Furthermore, no decline in this sensitivity with age was found for participants up to 60 years old. In contrast, sensitivity to tactile time differences across the wrists was found to be far worse than would be required to transfer across-ear time difference cues.

### How Well Are CI and Haptic Signals Combined in the Brain?

Anatomical, physiological, and behavioral studies all indicate that audio and haptic signals are strongly linked in the brain. Anatomical and physiological studies have revealed extensive connections from somatosensory brain regions at numerous stages along the auditory pathway, from the first node (the cochlear nucleus) to the cortex (Aitkin et al., 1981; Foxe et al., 2000; Shore et al., 2000, 2003). Physiological studies have also shown that substantial populations of neurons in the auditory cortex can be modulated by haptic stimulation (Lakatos et al., 2007; Meredith and Allman, 2015). Behavioral studies have demonstrated that haptic stimulation can affect auditory perception. Haptic stimulation has been found to facilitate the detection of faint sounds (Schurmann et al., 2004) and to modulate loudness and syllable perception (Gillmeister and Eimer, 2007; Gick and Derrick, 2009). Recent studies using EHS (reviewed above) have also shown that haptic stimulation can be integrated to improve sound localization (Fletcher et al., 2020a) and speech-in-noise performance (Huang et al., 2017; Fletcher et al., 2018, 2019, 2020b).

Given that audio and haptic signals can be integrated in the brain, it is important to understand how this integration can be maximized to increase EHS benefit. One important principle of multi-sensory integration is the principle of inverse effectiveness (Wallace et al., 1996; Hairston et al., 2003; Laurienti et al., 2006). This states that maximum multisensory integration occurs when senses provide low-quality information in isolation. This condition would appear to be well met in previous EHS studies, where participants received incomplete speech or sound location information through both their CI and through haptic stimulation. Another important principle for maximizing integration is correlation of temporal properties (Ernst and Bulthoff, 2004; Fujisaki and Nishida, 2005; Burr et al., 2009; Parise and Ernst, 2016). Again, this condition would appear to be well met in many EHS studies, where both audio and haptic signals were temporally complex and highly correlated.

### A Place for a New Generation of Tactile Aids?

Following from earlier work with tactile aids, modern haptic devices might be used to assist those who could benefit from a CI but cannot access or effectively use one. It is estimated that around 2% of CI users become non or minimal users (Bhatt et al., 2005; Ray et al., 2006). A higher proportion of non-use is found among adult CI recipients who were born deaf or who became deaf early in childhood (Lammers et al., 2018). Additionally, some deafened individuals achieve no or minimal benefit from a CI, for example, when cochlear ossification has occurred following meningitis (Durisin et al., 2015). Haptic technology has the potential to provide benefit to sound detection, discrimination, and localization, as well as speech perception in these groups. It could also benefit the many millions of people around the world who do not have access to hearing-assistive technologies, such as CIs, because of inadequate health-care provision or overburden some cost (Bodington et al., 2020; Fletcher, 2020).

All CI recipients undergo a period of auditory deprivation following surgery, as hearing aid use is not possible directly after implantation. For those undergoing bilateral implant surgery (which includes the majority of children receiving a CI in the United Kingdom), this can mean complete loss of auditory stimulation for a period of up to a month between CI surgery and initial device tuning. Another group that have a period of no or limited access to auditory stimulation are the 1–2% of CI users per year that experience device failure (Causon et al., 2013). These individuals typically face a wait of many months between the failure occurring and switch-on of a re-implanted device. Haptic stimulation could provide a means to maintain access to auditory information, including enhancing lip-reading, for these groups during this period of auditory deprivation. The effectiveness of haptic stimulation in supporting lip-reading has already been demonstrated in work using tactile aids (Kishon-Rabin et al., 1996).

Recent advances in key technologies provide an opportunity to develop a new generation of haptic aids that give greater benefit and have higher acceptance than the tactile aids of the 1980s and 1990s. Particularly important are advances in micro-motor, micro-processor, wireless communication, and battery technology, as well as in manufacturing and prototyping techniques such as 3D printing. These technologies will allow modern haptic devices to avoid many of the pitfalls of early tactile aids, such as bothersome wires, large power and computing units, highly limited signal-processing capacity, and short battery lives (for a detailed review of haptic device design considerations see Fletcher, 2020). Battery and wireless technology and improved manufacturing techniques will also reduce many of the practical and esthetic issues faced by earlier haptic devices. For example, new devices would not require wires to connect device components (e.g., microphones, battery and signal processing units, and haptic motors), can be more compact and discreet, and would require far less regular battery charging. In addition, modern haptic devices can deliver haptic signals with higher precision and deploy cutting-edge signal-processing techniques to substantially improve auditory feature extraction, particularly in the presence of background noise. Finally, modern haptic devices could improve safety and awareness by interfacing with smart devices in the internet of things, such as doorbells, telephones, and intruder or fire alarms.

Recently, haptic devices have been developed that—with further development—could likely be deployed as effective haptic aids to hearing. The mosaicOne_B (Fletcher et al., 2020c) is worn as a sleeve (15 cm long), with a total of 12 motors arranged along the dorsal and palmar sides of the forearm. The mosaicOne_C (Fletcher, 2020; see Figure 3), Tabsi (Pezent et al., 2019), and Buzz (Perrotta et al., 2021) are all wrist-worn devices, with multiple motors arranged around the wrist. In addition to delivering vibration, the Tabsi device includes a mechanism for modulating the amount of pressure (“squeeze”) applied to the wrist. Each of these devices use motor and haptic driver technology that overcomes many of the substantial haptic signal reproduction issues faced by earlier tactile aids (Summers and Farr, 1989; Cholewiak and Wollowitz, 1992). One haptic motor design (used in the mosaicOne_B) is the eccentric rotating mass, in which an asymmetric mass is turned to create vibration. These motors are low cost and able to produce high vibration intensity. However, they have quite low power efficiency, which limits their utility for real-world use. The vibration frequency and intensity of these motors change together and cannot be controlled independently. While this may be a limiting factor, it may also be advantageous for effective transfer of high-resolution information as higher sensitivity to change has been observed when frequency and intensity are modulated together than when either are modulated alone (Summers et al., 2005). Another low-cost motor design (used in the Tasbi and Buzz) is the linear resonant actuator, in which a mass is moved by a voice coil to create vibration. Linear resonant actuators are often unable to produce intense vibration but are highly power efficient. Unlike eccentric rotating mass motors, they operate at a single fixed frequency. A final alternative is the piezoelectric motor design, in which vibration is created by a material that bends and deforms as voltage is applied. Piezoelectric motors are able to produce complex waveforms (with the capacity to control the frequency spectrum and intensity independently) and are power efficient. However, they are currently typically much more expensive than linear resonant actuators or eccentric rotating mass motors.

The mosaicOne_B, mosaicOne_C, and Tasbi haptic devices are lab-based prototypes, with the haptic signal fed to the device through a separate unit that manages signal processing and audio capture. The Buzz, on the other hand, is available for real-world use. However, as discussed in Fletcher (2020), there are a number of important limitations in its current design. These include the capture of audio from an onboard microphone that is highly susceptible to wind noise and disruption from movement of clothing across the device. Previous EHS studies have advocated streaming of audio from behind-the-ear hearing-assistive devices, which already include technologies to address many of the issues faced by the Buzz (e.g., wind noise; Fletcher, 2020; Fletcher and Zgheib, 2020; Fletcher et al., 2020a, 2021a). This approach would also allow access to spatial-hearing cues and would increase the correspondence between audio and haptic stimulation, facilitating maximal multisensory integration. This approach could be readily implemented using existing wireless streaming technology (such as Bluetooth Low Energy), which is already implemented in the latest hearing-assistive devices. Alternatively, audio could be streamed from a remote microphone close to sound source of interest to maximize the signal-to-noise ratio (e.g., Dorman and Gifford, 2017). This may be particularly effective in noisy environments, such as classrooms.

## Areas for Further Investigation

### Benefit for Different Clinical Populations

It will be important for future work to establish how much EHS benefit can be achieved in different clinical populations. No study has yet aimed to compare EHS benefit across user groups. So far, EHS enhancement of speech-in-noise performance has been shown in unilaterally implanted CI users (Huang et al., 2017; Fletcher et al., 2019, 2020b) and in one bilaterally implanted participant [P9 in Fletcher et al. (2019)] for whom there was a large benefit (20.5% more words in noise recognized with EHS than with their CIs alone). A recent study that demonstrated EHS benefit to sound localization included only unilateral CI users, with around half also having a hearing aid in the non-implanted ear (Fletcher et al., 2020a). Although those without hearing aids benefitted most from EHS, substantial benefit was shown for both sets of participants.

Future work should also compare EHS benefit in those with congenital, early, and late deafness. Studies that have assessed multisensory integration in CI users have shown evidence that CI recipients with late-deafness and those with congenital or early-deafness who are implanted early are able to effectively integrate audio and visual information (Bergeson et al., 2005; Schorr et al., 2005; Tremblay et al., 2010). However, those implanted late (after a few years of deafness) integrate audio and visual information less effectively. Studies in non-human animals have also shown that extensive sensory experience in early development is required for multisensory integration networks to fully develop (Wallace and Stein, 2007; Yu et al., 2010). Although congenitally deaf CI recipients are able to effectively integrate audio and haptic information, some studies suggest that they do so less effectively than late-deafness CI recipients (Landry et al., 2013; Nava et al., 2014). This might suggest that congenitally deaf individuals will benefit less from EHS. However, there is also some evidence to suggest that congenitally deaf individuals have increased tactile sensitivity (Levanen and Hamdorf, 2001) and faster response times to tactile stimuli (Nava et al., 2014). This could mean that congenitally deaf people can access more information through haptic stimulation than those with late deafness and will therefore benefit more from EHS.

EHS benefit should also be assessed across different age groups. For haptic stimulation, like for hearing, detection and frequency-discrimination thresholds (particularly at high frequencies) worsen with age (Verrillo, 1979, 1980; Moore, 1985; Stuart et al., 2003; Reuter et al., 2012; Valiente et al., 2014). The ability to discriminate haptic stimulation at different locations on the skin has also been found to worsen with age (Leveque et al., 2000). However, intensity discrimination both at a single stimulation site (Gescheider et al., 1996a) and across sites (Fletcher et al., 2021a) has been found to be robust to aging. The evidence of decline in some aspects of haptic performance might suggest that EHS benefit will be reduced in older populations. However, the ability use haptic stimulation to achieve high sound-localization accuracy and enhanced speech-in-noise performance has been shown in both young (Fletcher et al., 2018; Fletcher and Zgheib, 2020) and older (Fletcher et al., 2019, 2020a, 2020b) adults. Furthermore, a range of evidence suggests that multisensory integration is increased in older adults (Laurienti et al., 2006; Diederich et al., 2008; de Dieuleveult et al., 2017), which could mean that EHS will be more effective in older CI users. In children, there may also be enhanced multisensory integration. One popular theory of brain development posits that infants are sensitive to a broad range of stimuli before becoming more specialized (a process known as “perceptual narrowing”; Slater and Kirby, 1998; Kuhl et al., 2006; Lewkowicz and Ghazanfar, 2009). There is evidence that a similar process occurs for multisensory integration (Lewkowicz and Ghazanfar, 2006). This could mean that EHS will be most effective in children, who have high tactile sensitivity and whose brains are most able to integrate novel multisensory stimuli.

### Ecologically Relevant Outcome Measures

To comprehensively assess EHS benefit, further testing with ecologically relevant outcome measures is required. This should include assessing EHS effects on speech prosody perception (rhythm, tone, intonation, and stress in speech) and listening effort. Speech prosody allows a listener to distinguish emotions and intention (e.g., the presence of sarcasm), and to distinguish statements from questions and nouns from verbs (e.g., “**ob**ject” from “ob**ject**”). CI users typically have impaired speech prosody perception (Xin et al., 2007; Meister et al., 2009; Everhardt et al., 2020) and report high levels of listening effort (Alhanbali et al., 2017). Access to pitch information has been shown to be critical to perception of speech prosody (Murray and Arnott, 1993; Banse and Scherer, 1996; Most and Peled, 2007; Xin et al., 2007; Peng et al., 2008; Meister et al., 2009). The mosaicOne_B haptic device, which was recently shown to transmit high-resolution pitch information (Fletcher et al., 2020c), would therefore appear a strong candidate device for recovering speech prosody perception in CI users.

Studies have so far shown that haptic stimulation can be used to accurately locate a single sound source (Fletcher and Zgheib, 2020; Fletcher et al., 2020a). It has also been shown that EHS improves speech recognition both for co-located and spatially separated speech and noise sources (Huang et al., 2017; Fletcher et al., 2018, 2019, 2020b). Future work should establish the robustness of haptic sound-localization to the presence of multiple simultaneous sounds and the extent to which EHS can enhance speech recognition in more complex acoustic environments, with numerous simultaneous sources at different locations.

### The Optimal Signal-Processing Strategy

To maximize EHS benefit, it will be critical to establish which sound features are most important for enhancing CI listening, and the most effective way to map these features to haptic stimulation. As already discussed, to date, most studies with EHS or tactile aids have focused on either *F_0* or speech amplitude envelope, but the effectiveness of presenting other sound features, such as spectral flatness or spread, either in addition or instead of these cues should also be explored. It will also be important to establish which noise reduction and signal enhancement strategies are most effective. As argued above, there is already a strong indication that an expander can be effective in allowing EHS to give benefit to speech-in-noise performance for co-located speech and noise sources (Fletcher et al., 2018, 2019, 2020b). However, more advanced noise-reduction techniques for enhancing speech-in-noise performance (e.g., Goehring et al., 2019; Keshavarzi et al., 2019) and music perception (e.g., Tahmasebi et al., 2020) should also be trialed, as well as techniques for enhancing spatial-hearing cues (Francart et al., 2011; Brown, 2014).

In addition to determining the optimal signal extraction strategy, the importance of individual tuning of the haptic device should be explored. Substantial additional EHS benefit might be achieved if haptic devices are, for example, effectively tuned to the individual’s tactile sensitivity (as in Fletcher and Zgheib, 2020), amount of residual acoustic hearing, or the CI device type or fitting used. It may also be important to adjust devices depending on how tightly the individual has secured the haptic device to their body, as this will affect the coupling of the haptic motor with the skin. This could involve exploiting existing methods, or those currently under development, which allow automatic correction for the amount of pressure applied to each motor in a device (Dementyev et al., 2020).

Another crucial consideration is how much time delay between audio and haptic signals can be tolerated while maintaining EHS benefit. This will dictate the sophistication of signal processing that can be used in EHS devices. One study explored the influence of haptic stimulation (air puffs) on the perception of aspirated and unaspirated syllables, with different delays between the audio and haptic signals (Gick et al., 2010). They found no significant change in the influence of haptic stimulation when it arrived up to 100 ms after the audio. This suggests that delays of several tens of milliseconds may be acceptable without reducing EHS benefit. A haptic signal can be delayed from an audio signal by up to around 25 ms before the signals are no longer perceived to be simultaneous (Altinsoy, 2003). This may suggest a delay of only a few tens of milliseconds would be tolerated. However, there is significant evidence that the brain rapidly corrects for consistent delays between correlated sensory inputs so that perceptual synchrony is retained (referred to as “temporal recalibration”; Navarra et al., 2007; Keetels and Vroomen, 2008; Van der Burg et al., 2013). If haptic stimulation can be delayed by several tens of milliseconds without reducing EHS benefit, this would allow for highly sophisticated signal-processing strategies to be implemented.

### The Neuroanatomical Basis of Electro-Haptic Enhancement

It will be important to understand how and where along the auditory pathway haptic and audio information are combined. One study of audio-tactile integration found somatosensory input was able to modulate the rhythm of ambient neural oscillations in auditory cortex. These oscillations were shifted into an ideal rhythm for enhancing auditory cortical responses to the auditory input (Lakatos et al., 2007). This may describe a key neural mechanism through which EHS enhances CI listening. In better understanding the mechanism, we might better understand how to maximize audio-tactile integration. This could inform how and where haptic stimulation is delivered, the choice of signal-processing approach, the design of training programs, and when in the CI care pathway EHS is introduced.

### Training

For EHS benefit to be maximized, optimal training regimes will need to be devised. EHS benefit has been shown to increase with training, both for enhancing speech-in-noise performance (Fletcher et al., 2018, 2019, 2020a) and for enhancing sound localization (Fletcher and Zgheib, 2020; Fletcher et al., 2020a). Earlier studies with tactile aids have also established that participants continue to improve their ability to identify speech presented through haptic stimulation (without concurrent audio) after months or even years of training (e.g., Sherrick, 1984; Brooks et al., 1986a, b; Weisenberger et al., 1987). So far, EHS studies have given only modest amounts of training and used simple training approaches. With more extensive training and more sophisticated training regimes, it seems likely that EHS can give even larger benefits than have already been observed.

## Conclusion

Haptic aids for the hearing-impaired were rendered obsolete in the 1990s by the development and success of CIs. However, researchers have recently shown compelling evidence that haptic stimulation can augment the CI signal, leading to enhanced speech-in-noise performance, sound localization, and music perception. Furthermore, significant developments in technology mean that the time is right for a new generation of haptic devices to aid the large number of people who are unable to access or benefit from a CI, whether for biomedical reasons or because of inadequate healthcare provision. With investment in the development of a low-power, compact, inexpensive, and non-invasive haptic device, the EHS approaches that have recently shown great promise in laboratory studies could soon be made available for testing in real-world trials. This new technology could enhance communication and quality of life for the nearly one million individuals who use CI technology, as well as the many millions of people across the world with disabling deafness who cannot access hearing-assistive devices.

## Author Contributions

MF drafted the manuscript. MF and CV edited and reviewed the manuscript. Both authors contributed to the article and approved the submitted version.

## Conflict of Interest

The authors declare that the research was conducted in the absence of any commercial or financial relationships that could be construed as a potential conflict of interest.

## Footnotes

1 The term “electro-haptic stimulation” is preferred to the term “electro-tactile stimulation” (which has also been used), as electro-tactile stimulation is commonly used to refer to electrical stimulation of the skin, rather than to haptic augmentation of CI listening.
